# Improved de-identification of physician notes through integrative modeling of both public and private medical text

**DOI:** 10.1186/1472-6947-13-112

**Published:** 2013-10-02

**Authors:** Andrew J McMurry, Britt Fitch, Guergana Savova, Isaac S Kohane, Ben Y Reis

**Affiliations:** 1 Harvard Medical School Center for Biomedical Informatics, Boston, MA USA; 2 Children's Hospital Informatics Program at the Harvard-MIT division of Health Sciences and Technology, Boston, MA USA; 3 Center for Advanced Genomic Technology, Boston University, Boston, MA USA; 4 Research Computing, Partners Healthcare System, Information Technology, Boston, MA USA; 5 Countway Library of Medicine, Harvard Medical School, 10 Shattuck St 4th Floor, Boston, MA 02115, USA

**Keywords:** Natural language processing (L01.224.065.580), Confidentiality (I01.880.604.473.650.500), Pattern recognition automated (L01.725), Electronic Health Records (E05.318.308.940.968.625.500)

## Abstract

**Background:**

Physician notes routinely recorded during patient care represent a vast and underutilized resource for human disease studies on a population scale. Their use in research is primarily limited by the need to separate confidential patient information from clinical annotations, a process that is resource-intensive when performed manually. This study seeks to create an automated method for de-identifying physician notes that does not require large amounts of private information: in addition to training a model to recognize Protected Health Information (PHI) within private physician notes, we reverse the problem and train a model to recognize non-PHI words and phrases that appear in public medical texts.

**Methods:**

Public and private medical text sources were analyzed to distinguish common medical words and phrases from Protected Health Information. Patient identifiers are generally nouns and numbers that appear infrequently in medical literature. To quantify this relationship, term frequencies and part of speech tags were compared between journal publications and physician notes. Standard medical concepts and phrases were then examined across ten medical dictionaries. Lists and rules were included from the US census database and previously published studies. In total, 28 features were used to train decision tree classifiers.

**Results:**

The model successfully recalled 98% of PHI tokens from 220 discharge summaries. Cost sensitive classification was used to weight recall over precision (98% F10 score, 76% F1 score). More than half of the false negatives were the word “of” appearing in a hospital name. All patient names, phone numbers, and home addresses were at least partially redacted. Medical concepts such as “elevated white blood cell count” were informative for de-identification. The results exceed the previously approved criteria established by four Institutional Review Boards.

**Conclusions:**

The results indicate that distributional differences between private and public medical text can be used to accurately classify PHI. The data and algorithms reported here are made freely available for evaluation and improvement.

## Background

Physician's notes contain information that may never be recorded in a coded format in the patient health record [1-3], such as family history [4], smoking history [5-7], and descriptions of lab results [8,9]. Nonetheless, the “uncoded” information buried in physician notes is so valuable that numerous attempts have been made towards indexing and sharing notes for research use. However, since physician notes can contain patient names, home addresses, social security numbers, and other types of Protected Health Information (PHI) [10], vast quantities of doctors’ notes have gone largely unused for medical research studies. Methods to simultaneously protect patient privacy and increase research utility are needed – as the number of electronic health record systems increases and with it the opportunity to study larger numbers of patients [11-13].

Existing methods for de-identifying medical texts range from simple rule-based systems to sophisticated machine learning algorithms [14,15]. The majority of currently implemented methods are rule-based systems that match patterns and dictionaries of expressions that frequently contain PHI [16]. The advantage of rule-based systems is that experts can quickly define rules and iteratively fine tune them to achieve higher accuracy. While rule-based systems have shown high recall in some settings [16], they often have the disadvantage of hard coding rules to a specific note format or physician writing style, resulting in poor performance in other contexts. Adjusting existing rule systems for use at other medical centers is often too costly, limiting broad use across institutions. This problem is well recognized [14,15], and has prompted efforts using an alternative, machine learning approach. Rather than using the expert to author rules, the rules for PHI removal are “learned” by training an algorithm using human annotated examples (i.e. a supervised learning task). For example, competitors in the i2b2 de-identification challenge [14] were asked to train or tune their algorithms on one set of human annotated notes and then validate their best model on a separate set of annotated notes. Generally, the highest scoring algorithms used machine learning methods such as conditional random fields [17-20], decision trees [21], support vector machines [22,23], and meta-classifiers that combine and weight different strategies for high-recall with false positive filtering [24].

The work reported here was trained and validated on the same i2b2 challenge datasets, which allows for comparison to prior work. Our algorithm performed favorably with regards to recall, albeit with lower precision (see results). The primary difference between our method and other top scores in the i2b2 challenge is the extensive use of publicly available medical texts to learn the distributional characteristics of individual PHI tokens that could appear in any type of physician note. This is in contrast to other models that use features that may be specific to the style and format of discharge summaries such as section headings [21], sentence position [18], and longer token sequences [17-20]. We show that publicly available medical texts provide an informative background distribution of sharable medical words, a property that is largely underutilized in patient privacy research.

## Methods

Instead of trying to recognize PHI words in physician notes, we reversed the problem towards recognizing non-PHI words. We asked, “what are the chances that a word or phrase would appear in a medical journal or medical dictionary? What are the lexical properties of PHI words? To what extent can we use publicly available data to recognize data that is private and confidential?”

While human annotated datasets of PHI are few in number and difficult to obtain, examples of public medical text are broadly available and generally underutilized for de-identification. By definition, medical journal publications provide the distributional evidence for words that are not PHI. Of course, some medical words will end up being proper names but the public corpora provide a heuristic measure of likelihood that we exploit as described below. In this context, relatively fewer human annotated examples are treated as approximations of the distributional properties of PHI. Lexical comparisons between PHI words and non-PHI words reveal that PHI words are generally nouns and numbers – whereas verbs and adjectives are probably ok to share -- especially medically relevant verbs and adjectives that are of more relevant to research studies. Publicly available lists of suspicious words and expert rules are also incorporated into this algorithm, such as US census data and the Beckwith regex list [16]. We combine the discrimination power of these complementary perspectives to achieve improved de-identification performance. As an additional safeguard, notes can be indexed [25] and later searched using coded medical concepts, thereby reducing the number of full-text reports that need to be shared in early phases of research [26].

### Design principles

**Figure 1:**
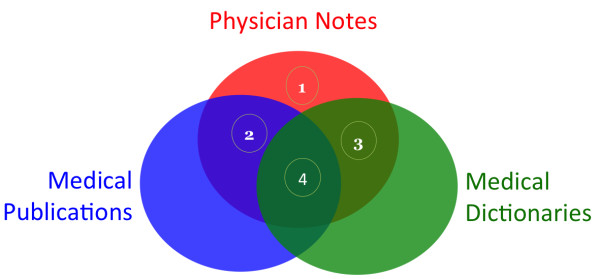
**Observations of physician notes with other types of medical text. 1)** Nouns and Numbers that only occur in physician notes are probably PHI. **2)** Words that occur frequently in medical publications are not likely to contain PHI. **3)** Words and phrases that occur frequently in medical dictionaries are not likely to contain PHI. **4)** Words shared in all three medical text sources are very unlikely to contain PHI.

The Scrubber was designed with the following general observations about physician notes and other types of medical text: (1) words that occur only in physician notes have increased risk for PHI, especially nouns and numbers which are the only types of PHI words; (2) words that occur in medical publications are not likely to refer to any specific patient; (3) words and phrases in medical vocabularies also do not refer to individually named patients; (4) words shared in many publically available medical text sources are very unlikely to contain PHI (Figure 1).

### Different types of PHI

The risk to patient confidentiality differs among the 8 major types of HIPAA defined PHI elements (Table 1). The acceptance criteria of the IRB focused on removing text that uniquely refers to a single patient including patient names, IDs such as medical record numbers, phone numbers, and home addresses. Fortunately, single patient identifiers are rarely necessary in patient research. As a secondary objective, this study sought to classify all types of PHI defined by HIPAA. This includes features that may refer to *many* patients, such as a hospital name, patient age, date of service, or doctor. These features are useful for studies of disease over time and should not necessarily be scrubbed if Limited Data Set [27] access is permitted by the hospital privacy board.

**Table 1:** Types of PHI and their risk to patient confidentiality

**PHI type**	**Minimum disclosure**	**Risk**
**Hospital**	**LDS**	**Minimal**
**Age**	**LDS**	**Minimal**
**Date**	**LDS**	**Minimal**
**Doctor**	**LDS**	**Minimal**
**Location**	**Identified**	**High**
**Patient**	**Identified**	**High**
**ID**	**Identified**	**High**
**Phone**	**Identified**	**High**

Minimum Disclosure refers to the level of permission typically required by an IRB. Limited Data Sets (LDS) contain features about a patient that refer to more than one patient. Identified level access almost always refers to a single patient, such as a patient name, medical record number, or phone number.

We anticipated that each type of PHI would have a unique set of association rules. For example, patient names are nouns whereas medical record numbers are numbers. Learning different association rules [28] for each type of PHI has the added benefit that additional weight can be placed on highest risk elements, such as the patient name or home address. All PHI types are generally represented as nouns and numbers with low term frequencies, low occurrence in medical controlled vocabularies, and non-zero regular expression matches of some type. Non-PHI words generally have higher term frequencies, higher occurrence in medical vocabularies and near zero matches in regular expressions of any type.

### Feature set construction

**Figure 2:**
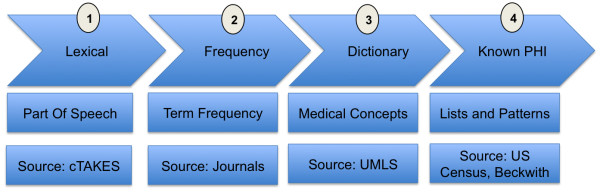
**Phases of the Scrubber annotation pipeline.** Lexical Phase: split document into sentences, tag part of speech for each token. Frequency Phase: calculate term frequency with and without part of speech tag. Dictionary Phase: search for each word/phrase in ten medical dictionaries. Known PHI Phase: match US census names and textual patterns for each PHI type.

The Scrubber pipeline constructs a feature set in four phases: lexical, frequency, dictionary, and known-PHI (Figure 2). First, the document instance is split into fragments and analyzed for part of speech and capitalization usage [29]. Second, term frequencies are assigned to each token in the document. Third, each fragment is matched against dictionaries of controlled medical vocabularies [30]. Lastly, US census data [31] and regular expression patterns are applied for each of the eight categories of PHI.

Apache cTAKES and Apache UIMA provide the foundation for the Scrubber pipeline. The data processing pipeline is provided by Apache UIMA project [32], an engineering framework commonly used in Natural Language Processing [33]. Of note, UIMA does not provide any pre-built components for text processing, it provides the main “scaffolding” and flow between user developed components. In the lexical phase, Apache cTAKES splits each document into sentences [34] and determines the part of speech for each token. Apache cTAKES is especially appropriate because it has been extensively trained on medical documents [29]. In the term frequency phase, the count of each token is retrieved from a corpus of open access medical publications previously annotated by Apache cTAKES. In the dictionary phase, each fragment is compared against phrases in publicly available sources, such as ICD9 diagnoses and LOINC laboratory concepts.

The annotation pipeline produces a high dimensional feature set that is very sparse, making the classification step more difficult. There are a number of ways to reduce dimensionality and increase feature set density, such as clustering similar features [35-37], removing features with low information content [38], reducing the number of class labels [28], and aggregating feature counts. Aggregating feature counts provided adequate feature density and reduced the dimensionality without discarding features that could be informative. Specifically, features were aggregated by source and type with respect to processing phase: lexical, frequency, dictionary, and known PHI (Table 2).

**Table 2:** Complete list of all 28 features annotated by the NLP pipeline

**Lexical**	**Frequency**	**Medical dictionary**	**Known PHI**
Part of Speech	Term Frequency (Token)	# matches HL7 2.5	# matches US Census Names
Part of Speech (Binned)	Term Frequency (Token, Part of Speech)	# matches HL7 3.0	
Capitalization		# matches ICD9 CM	# matches for pattern HOSPITAL
Word or Number		# matches ICD10 CM	# matches for pattern AGE
Length		# matches ICD10 PCS	# matches for pattern DATE
		# matches LOINC	# matches for pattern DOCTOR
		# matches MESH	# matches for pattern LOCATION
		# matches RXNORM	# matches for pattern PATIENT
		# matches SNOMED	# matches for pattern ID
		# matches COSTAR	# matches for pattern PHONE
		# consectutive tokens any dictionary	# consecutive tokens any pattern

In the lexical phase, part of speech and capitalization usage is annotated for each word token. In the frequency phase, each word is annotated with the frequency of appearance in public and private medical texts. In the dictionary phase, each word is compared to a list of standard medical concepts in UMLS sources. In the knownPHI phase, tokens and phrases are compared against suspicious patterns of HIPAA identifiers.

### Classification

The feature set is then processed through Weka [39] using a J48 decision tree [40] classification algorithm, a popular open source implementation of the C4.5 decision tree algorithm. J48 was chosen for several reasons. First, decision trees do not require “binning” value ranges to be effective [41]. This was useful because the correct value ranges were not known prior to classifier training. Second, decision trees can build a model for multiple class types. This is important because different types of PHI have different rules associated with them. For example, patient names are nouns whereas medical record numbers are numbers. A binary classifier would ignore these characteristic differences across PHI types and likely cause more errors.

### Training

The primary data used for training and testing was the I2B2 de-id challenge data [14]. This data consists of 669 training cases and 220 testing cases. The cases are a fully annotated gold standard set of discharge summaries. To calculate frequencies of word occurrences, we randomly selected 10,000 publicly available peer reviewed medical publications. This was necessary as many valid word tokens appear only once or not at all in any random selection of physician notes. Using more than 10,000 publications for training did not alter performance, and was computationally feasible using inexpensive commodity hardware.

On average there were 520 words (tokens) per case, and an average of 39 PHI words per case. As expected, most word tokens were not patient identifiers (PHI) -- the ratio of PHI words to non-PHI words was 1:15. Training a classifier using all of the available training instances would highly favor non-PHI classifications [42]. To address this issue, the training set was compiled using all of the PHI words and an equally sized random selection of non-PHI words.

## Results

### Summary

The training model was applied to an independent validation corpus of 220 discharge summaries from the i2b2 de-id challenge. The goal of this method is to produce a result meeting stated IRB requirements of high recall at the cost of precision. This method achieved its goal of very high recall (98%) and F10 (98%) albeit at the cost of a lower precision (62%) and F1 (76%). Compared to the seven participant groups in the original i2b2 de-identification challenge the scores reported in this paper would have placed first overall in recall and last overall in precision. The low precision is expected for several reasons. First, we did not tailor dictionaries and patterns for the i2b2 corpus and elected for an out-of-the-box approach which is more likely to mimic an initial deployment scenario. Secondly, our IRB stated that the most important metric for patient privacy is recall. In this context, recall is the percentage of confidential tokens that were removed relative to confidential tokens that were missed (Equation 1). Precision is the percentage of confidential tokens that were removed relative to the number of public tokens that were erroneously removed (Equation 2). The automated performance matches or exceeds that of two human evaluators [43] and preserves the readability of the original text [43].

Equation 1: Recall

$$\mathrm{recall}=\frac{\mathrm{number}\;\mathrm{of}\;\mathrm{confidential}\;\mathrm{tokens}\;\mathrm{removed}}{\mathrm{number}\;\mathrm{of}\;\mathrm{confidential}\;\mathrm{tokens}\;\mathrm{removed}+\mathrm{number}\;\mathrm{of}\;\mathrm{confidential}\;\mathrm{tokens}\;\mathrm{missed}}$$

Equation 2: Precision

$$\mathrm{precision}=\frac{\mathrm{number}\;\mathrm{of}\;\mathrm{confidential}\;\mathrm{tokens}\;\mathrm{removed}}{\mathrm{number}\;\mathrm{of}\;\mathrm{confidential}\;\mathrm{tokens}\;\mathrm{removed}+\mathrm{number}\;\mathrm{of}\;\mathrm{public}\;\mathrm{tokens}\;\mathrm{removed}}$$

### Classification accuracy

**Figure 3:**
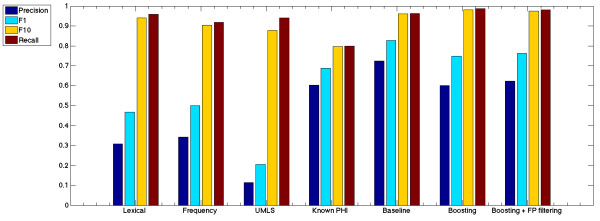
**Classifier results.** Lexical features include part of speech, capitalization usage, and token length. Frequency features refer to the token frequency across 10,000 medical journal publications. UMLS features refer to the number of matches for each token or phrase in ten medical dictionaries. Known PHI features include the US Census List and the patterns previously provided by Beckwith et al. Baseline classifier utilizes all feature groups using the J48 algorithm. Boosting used ten iterations of the Adaboost method. The false positive filter used in the final score to address potential false positives created during the boosting process.

**Figure 4:**
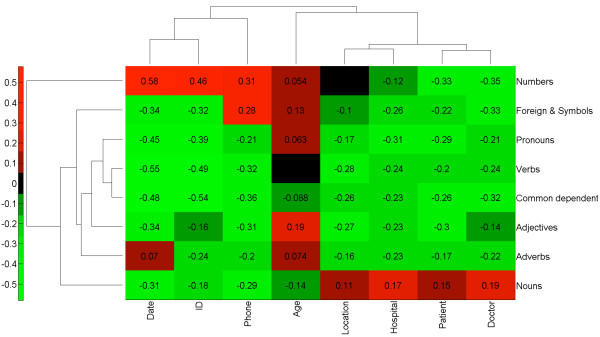
**Part of speech is highly informative for the PHI class.** Normalized pointwise mutual information was calculated between each Part Of Speech (POS) and PHI class. A score of 1 signifies the POS and PHI type always occur together. A score of −1 signifies the POS and PHI type never occur together. Clustering of the scoring matrix was calculated by Euclidean distance of the normalized scores. The results reveal that Nouns and Numbers have distinct groupings of PHI classes whereas all other parts of speech reduce the probability of any private PHI class.

Each feature group was trained and tested using a J48 classifier (Figure 3) using only the feature group specified in Figure 4. Lexical features include part of speech, capitalization usage, and token length. Frequency features refer to the token frequency across 10,000 medical journal publications. UMLS features refer to the number of matches for each token or phrase in ten medical dictionaries. Known PHI features include the US Census List and the patterns previously provided by Beckwith et al. [16]. All features were then included into a single baseline classifier using the J48 algorithm. Boosting was then performed to increase classifier recall of the minority PHI class types. Boosting was accomplished using ten iterations of the Adaboost method using default settings in Weka. Because boosting can lead to higher numbers of false positives, false positive filtering was then performed. The false positive filter was achieved by analyzing the 100 nearest neighbors of each token in the test set to ensure that all 100 neighbors in the training set have the same class label. If all 100 NN had the same class label, these samples were considered either background distribution of public words or very strong evidence of the PHI label.

### Misclassifications

The words “of”, “and”, “home”, “hospital”, and “services” were overwhelmingly the most commonly missed PHI words. These common words account for 124 of 173 partial misses, and pose little to no risk to patient privacy.

We performed a manual review of each misclassification and determined that no unique identifiers were left fully intact. Partial redactions – such as properly removing the patient last name but missing the patient first name were rare (13 word tokens in 12 cases). Lower risk identifiers such as hospital name and date of treatment were also rare. Only two dates and 2 hospital names were left fully intact.

### Lexical features

Every type of PHI is a noun or number (Figure 4). Interestingly, this fact alone yielded 96% recall (Figure 3). However, many naturally occurring words and medically relevant concepts can also appear as nouns and numbers. To distinguish PHI from nouns and numbers that are naturally occurring, a term frequency calculation was applied. Similarly, nouns and numbers with medical relevance were distinguished by their presence in one or more medical vocabularies.

### Term frequencies

Medical publications do not refer to individually named patients. Even in medical case studies, the patient name, home address, phone number, and medical record number must be withheld in accordance with law. This guarantees that all high-risk PHI elements in Table 1 will not be present in the publication dataset. It was therefore not surprising to find that patient specific identifiers were not frequently reported in the text of medical publications. As a result, classification of PHI using only term frequency features and part of speech yielded high scrubbing performance with 92% recall.

**Figure 5:**
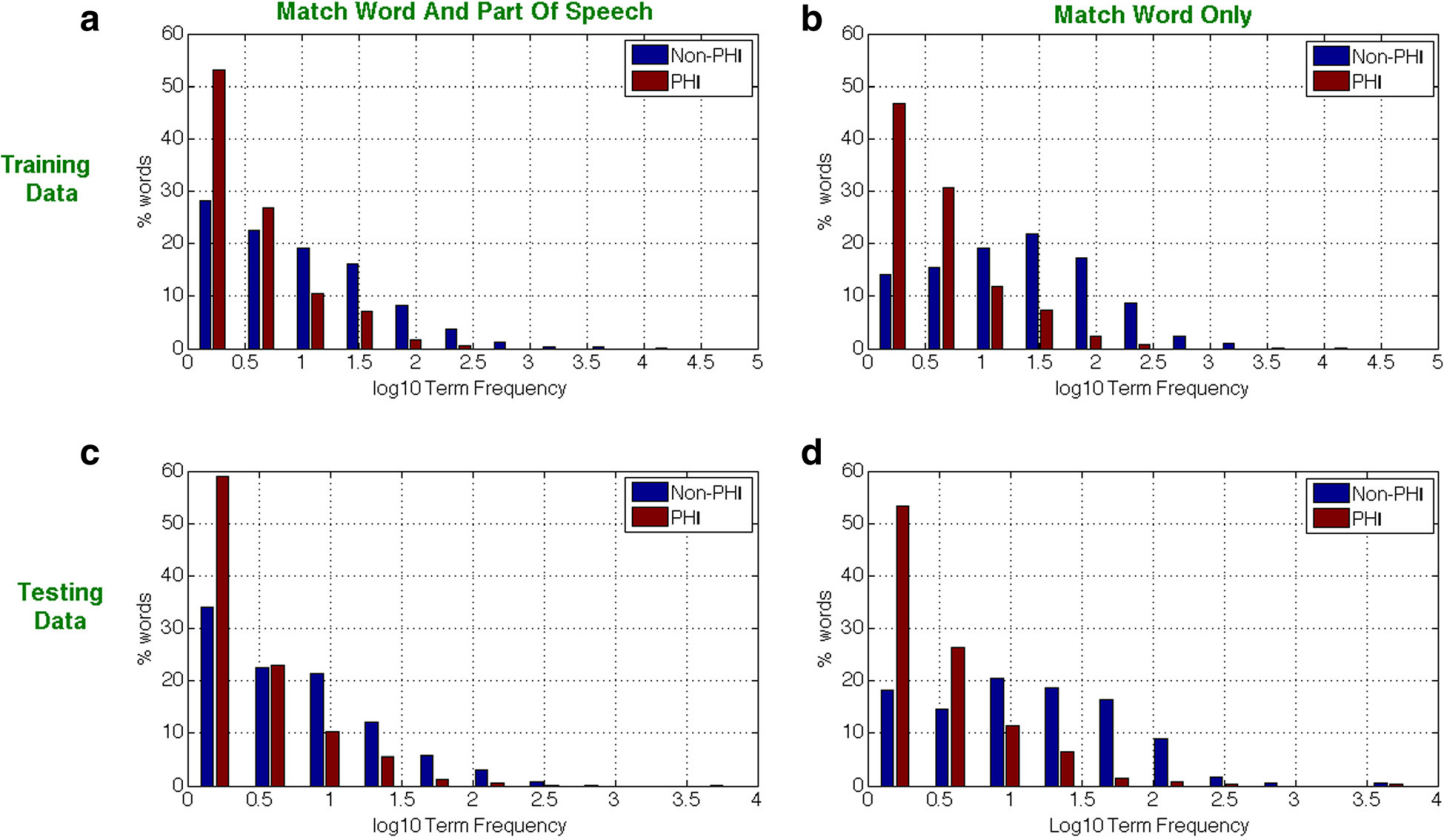
**Term frequency distributions in PHI and non-PHI word tokens.** In each of the four histograms, the log normalized term frequency (x-axis) is plotted against the percentage of word tokens. PHI words (red) are more common on the left hand side of each histogram, showing that PHI words tend to be rarer than non-phi words (blue). Top Figures **(a)** and **(b)** contain training data. Bottom Figures **(c)** and **(d)** contain testing data. Histograms for Training and Testing are characteristically similar. Term frequency histograms on the left **(a)** and **(c)** refer to words matched according to their part of speech. Term frequency histograms on the right **(b)** and **(d)** refer to raw word matches.

As expected, a first or last name would sometimes match an author name in the publication text. However, since author names and references list were removed during preprocessing, the overlap in names was minimized. There are other examples where patient identifiers can overlap with text in publications, for example when a patient lives on a street with the same name as a medical facility used in a published study. Nevertheless, patient identifiers are much less likely to appear in journal publications. To test and quantify this assumption, term frequencies were calculated across all word tokens in publication, training, and test datasets. Training and test datasets were split into groups of words containing PHI and not containing PHI. Histograms were then created, where the x-axis is the number of times a word appeared in all medical publications and the y-axis is the number of distinct words. A small percentage of common words created a skewed distribution, which was log normalized for visualization clarity. Figure 5 shows that PHI words are less frequently used in journal publications than non-PHI words. This is true with or without considering the part of speech for both the training and test datasets.

### Medical dictionaries

Ten vocabularies in the Unified Medical Language System were selected in order to span a very wide range of demographic terms, diagnoses, lab tests, medication names, and procedures. Surprisingly, a decision tree trained only to distinguish PHI from medical concepts yielded very high recall (94%), albeit with poor precision (12%). This suggests that there is almost no overlap between medical concepts and patient identifiers. These findings provide evidence that automatic retrieval of coded medical concepts (autocoding) is also useful for de-identification. In this way, parallel autocoding and de-identification provides maximum research utility while minimizing the risk of patient disclosure (Additional file 1: Table S2).

### Regular expressions

Regular Expressions yielded the most balanced ratio of recall (80%) to precision (60%) of any feature group tested in isolation (Figure 3). This matches our experience using a previous version of the HMS Scrubber in new medical center settings without customization and without inspecting the pathology report header [16]. We expected the regular expressions to outperform all other feature groups with respect to dates, phone numbers, and ages but this was not the case. This either means that we used Beckwith’s regular expression rules incorrectly or there are more ways to express these simple concepts than one might expect. Nevertheless, regular expressions slightly improved the overall classification specificity. The only changes to Beckwith’s regular expressions was the addition of one pattern for date, two for hospital names, and three for IDs.

### Quantifying the distance between public and private medical texts

Open access medical journals provide a heuristic measure of the words and phrases used to describe medical topics. Estimating the distributions of non-PHI tokens is therefore informative for recognizing PHI. To quantify this relationship, a vector space model [44-46] was created for journal publications and the i2b2 datasets. First, each dataset was annotated as described in “feature set construction”. Second, each numeric feature was normalized in the range between zero and one and mean subtracted. Third, the principal components were calculated for each part of speech (using defaults of Matlab pca method). Principal components were selected if they had >1 explained variance for a total explained variance of 99%. Fourth, the principal components were then compared in vector space by measuring the dot product (Equation 4).

Equation 3: F-measure

$$F_{\beta }=\left(1+\beta^{2}\right)*\frac{\mathrm{precision}*\mathrm{recall}}{\left(\beta^{2}*\mathrm{precision}\right)+\mathrm{recall}}$$

Equation 4: Vector similarity

$$\mathrm{sim}\left(d_{j},q\right)=\frac{d_{j}*q}{∥d_{j}∥∥q∥}$$

**Figure 6:**
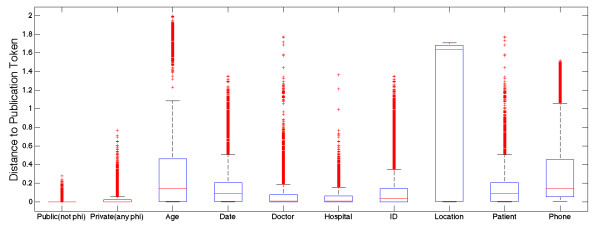
**Distance between tokens appearing in private and public medical texts.** Vector space model was used to capture the similarities (distance) in vector space between tokens in the private training set and tokens in the public publications set. In total, 669 physician notes and 669 medical publications were analyzed for their pairwise distances. An equal number of public (non-PHI) tokens and private tokens were selected from train. The boxplot shows 25th and 75th percentiles for distances from publication tokens to training tokens. The leftmost column reveals that public terms that are not PHI are more similar to publication tokens than any other group.

The similarity metric was then used to test the assumption that public texts provide a heuristic measure of the background distribution of non-PHI tokens (Figure 6). The overwhelming majority of public tokens from journal articles were more similar to non-PHI examples in the training set. This analysis revealed that confidential PHI tokens had far fewer similar examples than non-PHI tokens. We used this fact to create a false positives filtering component with a simple rule: if 100 nearest neighbors of a test token all had the same class label in the training set then that label is highly unlikely to be not PHI. In rare cases, the 100 nearest neighbors would all refer to the same type of PHI which we considered strong evidence for the PHI type.

### Validation

The i2b2 Challenge Data includes surrogate names that were constructed by permuting the syllables of real names in the US Census. This means that the provided names of patients, doctors, and hospitals are highly unlikely to be realistic examples, which could give an unnatural advantage to term frequency calculations and thus artificially improve classifier performance.

To verify that this was not the case, the i2b2 surrogate names were replaced with real names from the Medicare General Hospital Information file [47] and the US patent office list of US inventors [48]. Each hospital name in the i2b2 training and test datasets was replaced with a hospital name from Medicare. Each patient and doctor name in the i2b2 data was replaced with a randomly selected name from the list of US inventors. In total, Medicare provided 4838 unique hospital names and the USPTO provided 4301229 inventors (1473329 unique).

Validation results were very similar to the original results (Table 3).

**Table 3:** Classifier validation results

**Classifier validation**	**Precision**	**F1**	**F10**	**Recall**
Baseline	50	67	98	99
Boosted	59	74	98	99
Boosted + FP filtering	61	75	98	98

Validation was performed by replacing the i2b2 surrogate names with real names from Medicare and the US patent office. This was done to ensure that the model is not limited to the i2b2 surrogates. The results are similar to the original results.

## Discussion

Can we use vast quantities of public medical text to de-identify confidential information within private physician notes? Can we accelerate the rate of sharing physician notes for research without compromising patient confidentiality? Can we achieve these goals while respecting the challenges and responsibilities among hospital privacy boards? These questions motivated the authors to compare public and private medical texts to learn the distributions and lexical properties of Protected Health Information. The results of this experiment show that publicly available medical texts are highly informative for PHI recognition, resulting in performance that is likely to be approved for research use among by hospital review boards. The vast majority of misclassifications were common words appearing in hospital names, which pose minimal risk to patient privacy. A useful byproduct of this de-identification process is that coded medical concepts [49] are also stored for later search [26] and retrieval [50]. This approach to de-identification both reduces unauthorized disclosures and increases authorized use [51], a position previously confirmed by numerous hospital privacy boards [16,26,50].

Comparing public and private text sources reveals interesting properties of PHI. Words in physician notes that frequently appear in medical journal publications and concept dictionaries are highly unlikely to contain PHI. Conversely, words in physician notes that are nouns and numbers are more likely to contain PHI. It is interesting to speculate just how far publicly available text can be leveraged for de-identification tasks, and we encourage other researchers to use our annotated datasets and open source software for use in their own medical studies.

In a state of the art review of de-identification, Ozuner and Szolovits appropriately ask “how good is good enough? [14]” In this study, we sought to achieve performance levels that were already considered satisfactory by hospital privacy boards [16] with minimal investment. Numerous tradeoffs were made to achieve this goal. First, recall was strongly favored over precision, especially for patient names and ID numbers that have highest risk of disclosure. Second, we favored default configuration over hospital-specific human refinement. In our experience, site-specific modification of patient names lists and regular expressions can be laborious and can lead to “overscrubbing” information that is valuable for research. Third, we needed the algorithm to run on a single computer using commodity hardware, both to satisfy IRB concerns over data-duplication and reuse hardware already in place. Fourth, we wanted to make as few assumptions as possible about the training set to avoid unnecessary overfitting.

There were several limitations to this study. Term frequency calculations were performed for single word tokens. Increasing the term frequency to use two or more words might improve patient name recognition. For example, patients are more likely to have a first or last name in common with an author than a full name. Similarly, patient home addresses are highly unlikely to be found in published medical journals. However, common and rare word sequences can vary considerably across the different types and formats of physician notes and journal publications. We chose instead to err on the side of caution and use a single token model rather than ngrams or conditional random fields.

There is also the potential that we too have overfit our model to training examples and were fortunate enough to have the model validated in an independent sample. There are several cases where classifying PHI in new physician notes could be significantly less accurate. PHI words and phrases that frequently appear in medical publications and dictionaries are the most difficult to classify, although the number of times this occurs appears negligible. Incoherently written physician notes may be difficult to tag for part of speech, which would likely degrade classifier accuracy. Datasets that have different probability distributions and term frequency could also pose problems. In each of these potentially limiting examples, a new corpus would have to be characteristically different from the testing and training examples studied here.

We recommend that this de-identification method be used according to procedures that were previously acknowledged by four hospital IRBs [16,26]. The recommended workflow is as follows. Physician notes are de-identified and autocoded such that the scrubbed report is saved in a secured database and searchable according to medical vocabularies. Search access is limited to authorized investigators affiliated with the institution hosting the data, and under no circumstances should the textual data be made available for public download. Searching for patient cohorts matching study criteria occurs in an anonymized manner, meaning that only counts are returned with the first level of access. After finding a cohort of interest, an investigator may apply for access to review the deidentified cases. By increasing the level of access commensurate with the needs of a study [26], the risk to patient disclosure is minimized while allowing many investigators the ability to query and browse the valuable collection medical notes. The methods proposed here can be put to practical use today to help unlock the tremendous research potential of vast quantities of free-text physician notes accumulating in electronic medical record systems worldwide [13].

## Competing interests

Guergana K Savova is on the Advisory Board of Wired Informatics, LLC which provides services and products for clinical NLP applications.

Britt Fitch is a member of Wired Informatics, LLC which provides services and products for clinical NLP applications.

## Authors’ contributions

All authors made substantial contributions to the experimental design and result interpretation. AJM and BDF developed source code for the experiments. AJM, BDF, BR, GKS authored and revised the manuscript. All authors read and approved the final manuscript.

## Pre-publication history

The pre-publication history for this paper can be accessed here:

http://www.biomedcentral.com/1472-6947/13/112/prepub

## Supplementary Material

Additional file 1: Table S1 Part of Speech Binned. **Table S2.** Number of concepts per vocabulary listed in the UMLS. **Table S3.** Map of HIPAA defined PHI to Scrubber defined PHI. **Table S4.** Information Gain for i2b2 Challenge Data: original and validation datasets. Click here for file
