# Supplementary material for: Improved de-identification of physician notes through integrative modeling of both public and private medical text
Source: BMC Med Inform Decis Mak. 2013 Oct 2;13:112. doi: 10.1186/1472-6947-13-112 (PMC3907029; doi:10.1186/1472-6947-13-112)
Supplement: Additional file 1: Table S1 — Part of Speech Binned. Table S2. Number of concepts per vocabulary listed in the UMLS. Table S3. Map of HIPAA defined PHI to Scrubber defined PHI. Table S4. Information Gain for i2b2 Challenge Data: original and validation datasets. [file 1472-6947-13-112-S1.docx]

**Downloadable Open Access Data**

Annotated data sets can be downloaded from the Scrubber Open Source website. The annotations include the PHI class (non-PHI or one of the 8 PHI types) and 28 features including lexical, ontological, patterned, and statistical properties.

**Training and Evaluation**

The data used in this paper was from the i2b2 de-id challenge. This consisted of 2 sets of data:

1. TRAIN:
   1. 669 discharge summaries.
   2. Provided in both gold standard annotated form as well as unannotated.
2. TEST:
   1. 220 discharge summaries.
   2. Provided in both gold standard annotated form as well as unannotated.

In order to train a model or to evaluate how well the Scrubber algorithm performs on a given data set, we need to know which tokens are PHI and verify that after applying the model, we have correctly identified those tokens as PHI. This supplementary material should be sufficient for recreating the reported results. Additional programmer documentation and code is available on the Scrubber Open Source website (<https://open.med.harvard.edu/display/SCRUBBER/Software>).

The Scrubber is written in the Java language and uses several open source packages including UIMA, cTakes, and many processing libraries. The *KnownPHIExtractor* reads in i2b2 formatted annotated xml and determines the character position and content of the known PHI. This is stored in the database in the known_phi table for later comparision.

Alternatively, those preferring to train their own models and needing to annotate their own cases as training examples, are recommended to use the Protégé Ontology Editor and the Knowtator plugin which is a graphical user interface (GUI) for fast annotation of text sources according to some ontology (see section ‘Protégé/Knowtator Reference’ below for additional details regarding this third party software). If you use the Protégé application, you may export your annotations to the Protégé xml format and there is code provided in the Scrubber project in *KnownPHIExtractorProtege* to ingest this xml.

**Score Calculations**

The standard measures that we report are precision and recall. See Figure 3 in the main manuscript.

These measures are reported in three ways, based on the feature type (lexical, frequency, dictionary, known PHI), baseline algorithm, boosted algorithm, and boosted algorithm with false positive filtering. The purpose of reporting in this way is to show that the individual classifiers performed very poorly alone, but that combined they do a very good job at capturing PHI. In the practice of de-identification the correct capture of PHI is far more important than the sub classification of ‘Doctor’ vs. ‘Patient’ or ‘ID’ vs. ‘Date’.

**Results Details**

In total 186 PHI tokens were misclassified as the non-PHI class across 82 distinct cases.

13 misclassifications were PHI tokens that refer to a single patient. These misclassifications are 8 partial patient names, 4 partial patient locations, and 1 partial ID. These 13 misclassifications occur across 12 distinct cases.

173 misclassifications were PHI tokens that potentially refer to multiple patients and are generally considered lower risk than PHI tokens that refer to a single patient. These misclassifications are 5 partial hospital phone numbers, 1 partial hospital ID, 13 partial hospital locations, 1 partial date, 2 full dates, 15 partial doctor names, 2 full hospital names, and 134 partial hospital names (consisting of 111 instances of the word ‘of’ and 11 instances of other common partial names such as ‘hospital’, ‘clinic’, ‘and’, ‘home’, and ‘services’). These 173 misses occur across 75 distinct cases.

The literature suggests many different methods for counting hits and misses. Typically these are raw token counts, unique token counts,[[15](#_ENREF_15)] number of PHI instances (which may contain several tokens, for example a patient first and last name may make up 1 ‘patient’ instance), or only counting portions of PHI that identify the entity (for example, if the PHI entity were a hospital named ‘Massachusetts General Hospital’, it could be argued that missing ‘Hospital’ is not identifying, particularly because the instance tokens are replaced with a placeholder baring the class name such as ‘xxxHOSPITALxxx’.

We have taken a strict approach to assessing the efficacy of the Scrubber. If we miss the word ‘of’ or ‘hospital’ in an institution name this is counted against us even though the actual risk associated with this type of leakage is likely to be negligible.

In practice the risk associated with different types of PHI are not equivalent. PHI that refers to single patients such as patient names, IDs, and phone numbers pose a greater risk than PHI that refers to a doctor or an institution. We evaluated each of the 186 misclassifications to determine if the miss referred to an individual patient – suggesting high risk – or if the miss referred to an entity that is common across many patients – suggesting a lower risk. If considering only the number of tokens missed that referred to a single patient (13) we report a sensitivity of 99.9%.

**Part of Speech**

***Comparison of Medical Data Sets***

Part of speech tagging was applied to train and test data sets resulting in distributions that are characteristically similar. The train and test part of speech tags were then separated into PHI words and non-PHI words. This confirmed our assumption that PHI words are highly enriched for nouns and numbers and detectable using current methods available in cTAKES. Next, we examined the part of speech distribution for the 10,000 publications used to train the classification model. The distribution of part of speech in journal publications more closely resembles the distribution of non-PHI words in the train and test physician notes.

***Binning***

We used the raw Part of Speech (PoS) tag as well as a Binned PoS as features in our classifier. The binning process was straightforward and is displayed in the TABLE S1 below. The purpose of binning is to increase feature density, or rather to address data sparcity. For example, the adjective denoted by JJ is far more common than the comparative or superlative forms of JJR or JJS.

**Pipeline**

One of the goals of Scrubber 3.0 was to create a product that was very modularized and could be easily adapted to include new information without altering the structure in any significant way. For example, Soundex algorithms can be used effectively to identify many cases of misspelled PHI, which is a very common problem in de-identifcation. However, there are many shortcomings to Soundex, specifically that it performs very poorly on short words. By employing pipeline architecture we are able to plug-and-play new pipelines with only the most minor of edits. This makes trying new algorithms and adding features easier given the decoupled components.

**Sliding Window**

The DictionaryAnnotator uses a sliding window over all phrases. The purpose of the dictionary annotator is to lookup tokens or phrases from the medical text within controlled medical dictionaries. The purpose of the sliding window is to try to identify individual pieces of non-PHI as part of a larger piece of non-PHI that taken alone would not have been part of a controlled vocabulary.

For example, the noun phrase ‘insulin dependent diabetes mellitus’ will be queried against the controlled vocabularies (Table S2) using a forward and backward sliding window. That is, we will try to find matches for:

1. ‘insulin dependent diabetes mellitus’
2. ‘insulin dependent diabetes’
3. ‘insulin dependent’
4. ‘insulin’
5. ‘dependent diabetes mellitus’
6. ‘diabetes mellitus’
7. ‘mellitus’

The result is that we gain feature values for words that would otherwise have none. In this case, the term ‘mellitus’ does not appear in any of our vocabularies by itself, however, it does appear in the larger term ‘diabetes mellitus’, amongst others. This allows us additional information suggesting that these tokens are not PHI, based on their presence in controlled medical vocabularies.

**Publication Processing**

The publication set used was a sample of 10,000 randomly selected publications from the available set of open access publications from the NLM (<http://www.ncbi.nlm.nih.gov/pmc/tools/openftlist/>). There is code that handles parsing these documents (PubExtractor & PubParser). Before these pubs are parsed we apply some very simple regex (ReferenceTextStripper) to replace inline citations in the form “*Name* et al.” with “Author et al.” This is just to reduce the number of names present in the publication space.

**Protégé/Knowtator Annotation References**

1. Install Protégé 3.3: <http://protege.cim3.net/download/old-releases/3.3.1/basic/>
2. Download Knowtator 1.7: <http://sourceforge.net/projects/knowtator/files/Knowtator/Knowtator%201.7/>
3. Install Knowtator: <http://knowtator.sourceforge.net/install.shtml>
4. Quickstart guide for setting up a new project: <http://knowtator.sourceforge.net/quickstart.shtml>
5. General Documentation: <http://knowtator.sourceforge.net/docs.shtml>
6. Data Management: <http://knowtator.sourceforge.net/data-management.shtml#merge>
7. Memory Usage: <http://protegewiki.stanford.edu/wiki/Setting_Heap_Size>

**Running the Scrubber Algorithm**The Open Source implementation of the de-identification algorithm comes with instructions for installing and running the software. In the project under the *doc* directory there is a document named scrubber-3.x-runtime-guide.pdf. This document describes all steps necessary to reproduce our results. Most of the steps also supply shell scripts to make this process simpler.

**Supplemental Material Figures/Tables**

| **Part of Speech** | **Part of Speech Bin** |
| --- | --- |
| **FW** - Foreign word | **FW-Symb** |
| **SYM** - Symbol | **FW-Symb** |
| **JJ** - Adjective | **Adjectives** |
| **JJR** - Adjective, comparative | **Adjectives** |
| **JJS** - Adjective, superlative | **Adjectives** |
| **CD** - Cardinal number | **Numbers** |
| **LS** - List item marker | **Numbers** |
| **NN** - Noun, singular or mass | **Nouns** |
| **NNS** - Noun, plural | **Nouns** |
| **NNP** - Proper noun, singular | **Nouns** |
| **NNPS** - Proper noun, plural | **Nouns** |
| **CC** - Coordinating conjunction | **Common Dependent Words** |
| **DT** - Determiner | **Common Dependent Words** |
| **EX** - Existential there | **Common Dependent Words** |
| **IN** - Preposition or subordinating conjunction | **Common Dependent Words** |
| **MD** - Modal | **Common Dependent Words** |
| **PDT** - Predeterminer | **Common Dependent Words** |
| **RP** - Particle | **Common Dependent Words** |
| **TO** - to | **Common Dependent Words** |
| **UH** - Interjection | **Common Dependent Words** |
| **WDT** - Wh-determiner | **Common Dependent Words** |
| **VB** - Verb, base form | **Verbs** |
| **VBD** - Verb, past tense | **Verbs** |
| **VBG** - Verb, gerund or present participle | **Verbs** |
| **VBN** - Verb, past participle | **Verbs** |
| **VBP** - Verb, non-3rd person singular present | **Verbs** |
| **VBZ** - Verb, 3rd person singular present | **Verbs** |
| **PRP** - Personal pronoun | **Pronouns** |
| **PRP$** - Possessive pronoun (prolog version PRP-S) | **Pronouns** |
| **WP** - Wh-pronoun | **Pronouns** |
| **WP$** - Possessive wh-pronoun (prolog version WP-S) | **Pronouns** |
| **RB** - Adverb | **Adverbs** |
| **RBR** - Adverb, comparative | **Adverbs** |
| **RBS** - Adverb, superlative | **Adverbs** |
| **WRB** - Wh-adverb | **Adverbs** |
| **'** | **apos** |
| **,** | **comma** |
| **.** | **period** |
| **:** | **Unknown** |
| **()** | **paren** |

**Table S1: Part of Speech Binned.** Similar parts of speech were grouped to increase feature

set density. Nouns, pronouns, verbs, adverbs, and adjectives already have natural groupings.

Common dependent words, foreign words and symbols, and punctuation were each assigned

a unique category.

| Vocabularies | # Concepts |
| --- | --- |
| COSTAR | 3,461 |
| HL7V2.5 | 5,020 |
| HL7V3.0 | 8,062 |
| ICD10CM | 102,048 |
| ICD10PCS | 253,708 |
| ICD9CM | 40,491 |
| LOINC | 327,181 |
| MESH | 739,161 |
| RXNORM | 437,307 |
| SNOMEDCT | 1,170,855 |

**Table S2: Number of concepts per vocabulary listed in the UMLS.** 10 Vocabularies were selected in order to span a very wide range of demographic terms, diagnoses, lab tests, medication names, and procedures.

| HIPAA PHI | Scrubber PHI Binned |
| --- | --- |
| 1. Names; | PATIENT, DOCTOR, HOSPITAL |
| 2. All geographical subdivisions smaller than a State, including street address, city, county, precinct, zip code, and their equivalent geocodes, except for the initial three digits of a zip code, if according to the current publicly available data from the Bureau of the Census: (1) The geographic unit formed by combining all zip codes with the same three initial digits contains more than 20,000 people; and (2) The initial three digits of a zip code for all such geographic units containing 20,000 or fewer people is changed to 000. | LOCATION |
| 3. All elements of dates (except year) for dates directly related to an individual, including birth date, admission date, discharge date, date of death; and all ages over 89 and all elements of dates (including year) indicative of such age, except that such ages and elements may be aggregated into a single category of age 90 or older; | DATE, AGE |
| 4. Phone numbers; | PHONE |
| 5. Fax numbers; | PHONE |
| 6. Electronic mail addresses; | PHONE |
| 7. Social Security numbers; | ID |
| 8. Medical record numbers; | ID |
| 9. Health plan beneficiary numbers; | ID |
| 10. Account numbers; | ID |
| 11. Certificate/license numbers; | ID |
| 12. Vehicle identifiers and serial numbers, including license plate numbers; | ID |
| 13. Device identifiers and serial numbers; | ID |
| 14. Web Universal Resource Locators (URLs); | PHONE |
| 15. Internet Protocol (IP) address numbers; | PHONE |
| 16. Biometric identifiers, including finger and voice prints; | N/A |
| 17. Full face photographic images and any comparable images; and | N/A |
| 18. Any other unique identifying number, characteristic, or code (note this does not mean the unique code assigned by the investigator to code the data) | ID |

**Table S3: Map of HIPAA defined PHI to Scrubber defined PHI.**

| i2b2 Training Dataset (original) | |  | I2b2 Training Dataset (validation) | |
| --- | --- | --- | --- | --- |
|  |  |  |  |  |
| **InfoGain** | **Feature** | *Rank* | **InfoGain** | **Feature** |
| 0.26355 | Frequency.token | *#1* | 0.27464 | Frequency.token |
| 0.25609 | Frequency.token.with_part_of_speech | *#2* | 0.2657 | Frequency.token.with_part_of_speech |
| 0.21836 | KnownPHI.regex.max_tokens | *#3* | 0.20706 | Lexical.ctakes.chunker.part_of_speech |
| 0.17967 | Lexical.ctakes.chunker.part_of_speech | *#4* | 0.20427 | KnownPHI.regex.max_tokens |
| 0.16957 | KnownPHI.regex.count.date | *#5* | 0.16935 | KnownPHI.regex.count.date |
| 0.14549 | Lexical.ctakes.**c**hunker.part_of_speech.bin | *#6* | 0.15124 | Lexical.ctakes.chunker.part_of_speech.bin |
| 0.13205 | Lexical.ctakes.token.type | *#7* | 0.13207 | Lexical.ctakes.token.type |
| 0.07965 | KnownPHI.regex.count.ID | *#8* | 0.08734 | Lexical.ctakes.WordToken.capitalization |
| 0.07629 | Lexical.ctakes.WordToken.capitalization | *#9* | 0.07944 | KnownPHI.regex.count.ID |
| 0.06354 | Lexical.ctakes.token.length | *#10* | 0.06156 | Lexical.ctakes.token.length |
| 0.0516 | Dictionary.umls.max_consecutive_tokens | *#11* | 0.05719 | Dictionary.umls.max_consecutive_tokens |
| 0.04255 | Dictionary.umls.count.SNOMEDCT | *#12* | 0.04801 | Dictionary.umls.count.SNOMEDCT |
| 0.04108 | KnownPHI.regex.count.doctor | *#13* | 0.0428 | KnownPHI.regex.count.doctor |
| 0.04012 | KnownPHI.regex.count.hospital | *#14* | 0.03446 | Dictionary.private.dictionary |
| 0.02841 | KnownPHI.regex.count.phone | *#15* | 0.02836 | KnownPHI.regex.count.phone |
| 0.02531 | Lexical.ctakes.token.PunctuationToken | *#16* | 0.02704 | Lexical.ctakes.token.PunctuationToken |
| 0.02222 | Dictionary.umls.count.LNC | *#17* | 0.02553 | Dictionary.umls.count.LNC |
| 0.01695 | Dictionary.umls.count.MSH | *#18* | 0.01941 | Dictionary.umls.count.MSH |
| 0.01499 | Dictionary.umls.count.HL7V3.0 | *#19* | 0.01618 | Dictionary.umls.count.HL7V3.0 |
| 0.01298 | Dictionary.umls.count.HL7V2.5 | *#20* | 0.01492 | Dictionary.umls.count.HL7V2.5 |
| 0.00695 | Dictionary.private.dictionary | *#21* | 0.01211 | KnownPHI.regex.count.hospital |
| 0.00441 | Dictionary.umls.count.COSTAR | *#22* | 0.00496 | Dictionary.umls.count.COSTAR |
| 0.00246 | Dictionary.umls.count.RXNORM | *#23* | 0.00304 | KnownPHI.regex.count.patient |
| 0.00218 | Dictionary.umls.count.ICD9CM | *#24* | 0.00247 | Dictionary.umls.count.RXNORM |
| 0.00216 | KnownPHI.regex.count.patient | *#25* | 0.00245 | Dictionary.umls.count.ICD9CM |
| 0.00152 | KnownPHI.regex.count.address | *#26* | 0.00137 | KnownPHI.regex.count.address |
| 0.00147 | Dictionary.umls.count.ICD10CM | *#27* | 0.00123 | Dictionary.umls.count.ICD10CM |
| 0.00106 | KnownPHI.regex.count.age | *#28* | 0.00107 | KnownPHI.regex.count.age |

**Table S4: Information Gain for i2b2 Challenge Data: original and validation datasets.**

The original dataset refers to the complete set of i2b2 training examples with synthetic names.

The validation dataset refers to the complete set of i2b2 training examples with real names from Medicare and the US patent office. Information Gain is the standard metric to determine splits in decision trees. Information Gain is the measure of entropy in the class minus the conditional entropy (H(Class) - H(Class | Feature)). Each dataset was processed through Weka using Information Gain (Weka InfoGainAttributeEval, default settings).
